# Diseases of Women

**Published:** 1895-06

**Authors:** 


Diseases of Women. By Henry J. Garrigues, A. M., M. D., professor of obstetrics in the New York Post-Graduate Medical School and Hospital; gynæcologist to St. Mark's Hospital in New York City; gynæcologist to the German Dispensary in the city of New York; consulting obstetric surgeon to the New York Maternity Hospital, etc. Complete in one handsome royal octavo volume of 700 pages. Illustrated with 310 engravings and colored plates. Full and comprehensive index. Prices: Cloth, $4.00; sheep, $5.00 net. W. B. Saunders, publisher, 925 Walnut Street, Philadelphia.

The reception accorded to this work has been most flattering. In the short period which has elapsed since its issue, it has been adopted and recommended as a text-book by more than sixty of the medical schools and universities of the United States and Canada.

